# Cholesteric Liquid Crystal Polymeric Coatings for Colorful Artificial Muscles and Motile Humidity Sensor Skin Integrated with Magnetic Composites

**DOI:** 10.1002/adfm.202300731

**Published:** 2023-02-28

**Authors:** Wei Feng, Aniket Pal, Tianlu Wang, Ziyu Ren, Yingbo Yan, Yanqing Lu, Huai Yang, Metin Sitti

**Affiliations:** Physical Intelligence Department, Max Planck Institute for Intelligent Systems, 70569 Stuttgart, Germany; Physical Intelligence Department, Max Planck Institute for Intelligent Systems, 70569 Stuttgart, Germany; Physical Intelligence Department, Max Planck Institute for Intelligent Systems, 70569 Stuttgart, Germany; Institute for Biomedical Engineering, ETH Zürich, 8092 Zürich, Switzerland; Physical Intelligence Department, Max Planck Institute for Intelligent Systems, 70569 Stuttgart, Germany; Physical Intelligence Department, Max Planck Institute for Intelligent Systems, 70569 Stuttgart, Germany; Laboratory for Multiscale Mechanics and Medical Science, SV LAB, School of Aerospace, Xi'an Jiaotong University, Xi'an 710049, China; National Laboratory of Solid-state Microstructures, College of Engineering and Applied Sciences, Nanjing University, Nanjing 210093, China; Beijing Advanced Innovation Center for Materials Genome Engineering & School of Materials Science and Engineering, Peking University, Beijing 100871, China; Physical Intelligence Department, Max Planck Institute for Intelligent Systems, 70569 Stuttgart, Germany; Institute for Biomedical Engineering, ETH Zürich, 8092 Zürich, Switzerland; School of Medicine and College of Engineering, Koç University, Istanbul 34450, Turkey

**Keywords:** cholesterics, humidity-responsiveness, liquid crystal networks, magnetic actuations, structural colors

## Abstract

Structural colorful cholesterics show impressive susceptibility to external stimulation, leading to applications in electro/mechano-chromic devices. However, out-of-plane actuation of structural colorful actuators based on cholesterics and the integration with other stimulation remains underdeveloped. Herein, colorful actuators and motile humidity sensors are developed using humidity-responsive cholesteric liquid crystal networks (CLCNs) and magnetic composites. The developed colorful actuator can exhibit synergistic out-of-plane shape morphing and color change in response to humidity, with CLCNs as colorful artificial muscles. Through the integration with magnetic control, the motile sensor can be navigated to open and confined spaces with the aid of friction to detect local relative humidity. The integration of multi-stimulation actuation of cholesteric magnetic actuators will expand the research frontier of structural colorful actuators and motile sensors for confined spaces.

## 1 Introduction

Colorful outlooks have several benefits for living creatures, for example chameleons and octopi, in the purpose of camouflage, body protection, signaling, and physiology.^[1,2]^ Researchers have thus been inspired to develop artificial colorful devices to mimic natural counterparts.^[3–11]^ Coloration of these devices was usually realized by fluorescent elements, organic dye/pigments, or structural color.^[12–20]^ Fluorescent pigments require UV light to visualize while organic synthetic pigments are often with photobleaching problems. In contrast, structure color remains vivid under daylight and is free of photobleaching problems.

Most of the structural colors result from ordered microstructures, including scattering, thin-film interference, gratings, metasurfaces, total internal reflection, and photonic crystals.^[21–24]^ As a typical kind of photonic crystals, cholesteric liquid crystals consist of rod-like liquid crystalline mesogens and periodically align in a helical manner.^[25]^ Cholesterics reflect the light of certain wavelength following Bragg's Law: *λ* = npsin*θ*, where *λ* is the central wavelength of reflected light, n is the average refractive index of liquid crystals, p represents the pitch of cholesteric liquid crystals and *θ* is the incidence angle. Impressive progress has been made in the development of dynamic colorful cholesteric liquid crystal devices.^[26–31]^ However, most of the previously reported devices were in the form of static coatings/liquid crystal glass cells or polymer film sheets with 1D in-plane shrinkage/elongation. Despite impressive reports on the dynamic color change of the humidity-responsive CLCNs, attention was mainly focused on the chromatic response of CLCN coatings adhered to substrates.^[32–34]^ The mechanical deformation parameters of hygroscopic CLCNs that are critical for robotics remain unexplored, and 3D actuation remains rarely demonstrated. While for 3D actuators based on liquid crystal polymers,^[26,35–43]^ the sample cutting direction couples with the deformation modes (e.g, curling or twisting) since LC actuators usually adopted spatially non-uniform molecular configurations, e.g., radial/splay/twist-nematic alignment.^[39–43]^ This coupling complicates the fabrication of the actuator with complex shapes and desired actuation modes. Such molecular configurations are also colorless and could not be directly used to develop colorful actuators.

In the present study, we show the application of the humidity-responsive CLCN as a platform to develop colorful actuators with synergistic color change and shape morphing when the CLCNs act as colorful artificial muscles, and the control of actuators by humidity and magnetic field; we also showcase CLCNs' applications in robotic and motile sensing fields while CLCN coatings act as humidity sensors with the integration of magnetic actuation and aid of friction, since currently most of previously sensors were usually static. The aforementioned coupling between the actuation mode and actuator cutting direction is overcome by using cholesteric alignment where mesogens rotate along the helical axis/film thickness direction, which is accompanied by the benefit of endowing the actuator with structural color. We first utilize humidity-sensitive contractile CLCNs as colorful artificial muscles to develop structural colorful actuators with synergistic out-of-plane shape morphing and color change in response to external stimulus, i.e., humidity, and demonstrate the multi-stimuli robotic control using the magnetic field in addition to humidity. Specifically, we develop colorful actuators mimicking blooming flowers and butterfly flapping wings to show their synergistic shape morphing/color change performance under humidity and magnetic stimulation. We then show the application of magnetic actuation that could navigate motile sensors to different places and sense local humidity where commercial tethered sensors are not facile to implement.

## 2 Results and Discussion

### 2.1 Humidity-Responsive CLCN

**Figure 1:**
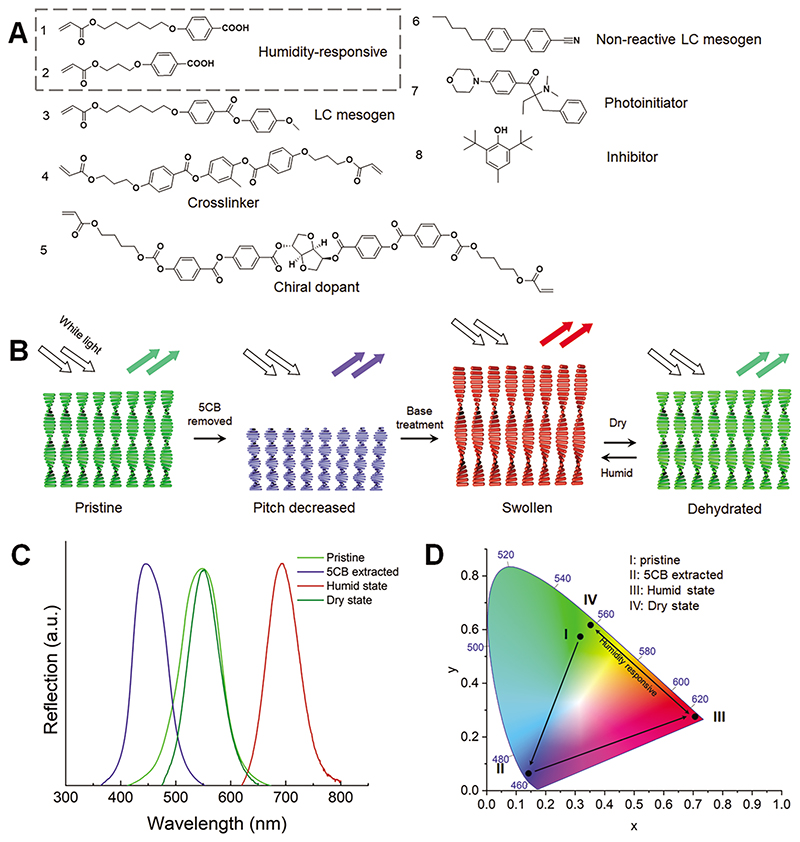
Humidity-responsive cholesteric liquid crystal networks (CLCN). A) Chemical structures of LC monomers, photoinitiator, and inhibitor. B) Schematic illustration of processing of humidity-responsive CLCN. Non-reactive 5CB was extracted from the film, leading to decrease in pitch. Base treatment of the CLCN deprotonated the carboxylic acid groups and made the CLCN hygroscopic. The cholesteric pitch and film thickness increased in response to humidity exposure. C) Corresponding reflection spectra of different states showed in B. D) Reflection band-correlated positions in the CIE-1931 color space.

CLCN was prepared via photopolymerization of a set of liquid crystal (LC) monomers (experimental details can be found in Experimental Section). Humidity-responsive carboxylic acid groups-containing monomers (Monomers 1 and 2 in Figure 1A) endowed the CLCN with humidity sensitivity. Monomers 3 (RM105) and 6 (5CB) were included to manifest a wide temperature range of cholesteric phase around room temperature from 71 to 22 °C (DSC curve in Figure S1, Supporting Information). Non-reactive LC mesogen 6 allowed a large tunable range in the reflection band. Cross-linker 4 (RM257) was used to form the cross-linked polymeric network, while chiral dopant 5 (LC756) promoted LC molecules to self-assemble into chiral helical structures and selectively reflect light of certain wavelength. The concentration of chiral dopant could be altered to change the helical pitch and obtain desired reflection spectrum following the equation *p* = 1/(*HTP*·*c*), where *HTP* and *c* are helical twisting power and concentration of the chiral dopant, respectively.^[44]^ Photoinitiator 7 (IRGACURE 369) was used to initiate photopolymerization while inhibitor 8 (Butylated hydroxytoluene, BHT) was used to prevent premature thermal-induced polymerization during sample processing. The compositions of monomers were optimized to afford a broad nematic phase and enable LCN to show wide color spectra change upon base treatment. To prepare LC films, the LC monomer mixture in isotropic phase at 80 °C was filled into the LC cell composed of two glass substrates with a gap of 20 μm that were coated with antiparallel-rubbed PVA. In the monomeric state, the cholesteric monomer mixture showed temperature-dependent optical reflection. The reflection band was red-shifted upon cooling (Figure S2, Supporting Information), and could be used to control the initial green color of the pristine polymerized LCN film by choosing a suitable polymerization temperature. Here we choose to photopolymerize at 37 °C to harvest pristine green LCN films with a reflection peak at 548 nm (Figure 1B,C).

After polymerization, unreacted 5CB was extracted from the CLCN film with dichloromethane, resulting blue shift of the reflection band attributed to the decreased pitch of cholesterics (Figure 1B,C). With the existence of hygroscopic carboxylate salt groups, the CLCN film showed an obvious response to humidity in terms of shape and light reflection. In detail, carboxylate salt groups absorbed water, resulting in swollen CLCN film and an increase in the cholesteric pitch. The reflection band was thus red-shifted and the film color changed from blue to red. Reversibly, water was desorbed in a dry environment, resulting in shrinkage of the cholesteric pitch and blue-shift of the reflection band (Figure 1C,D). The reflection band data-derived coordinates are located in corner positions of the CIE1931 color space (Figure 1D), indicating vivid and strong contrast structural colors.

**Figure 2:**
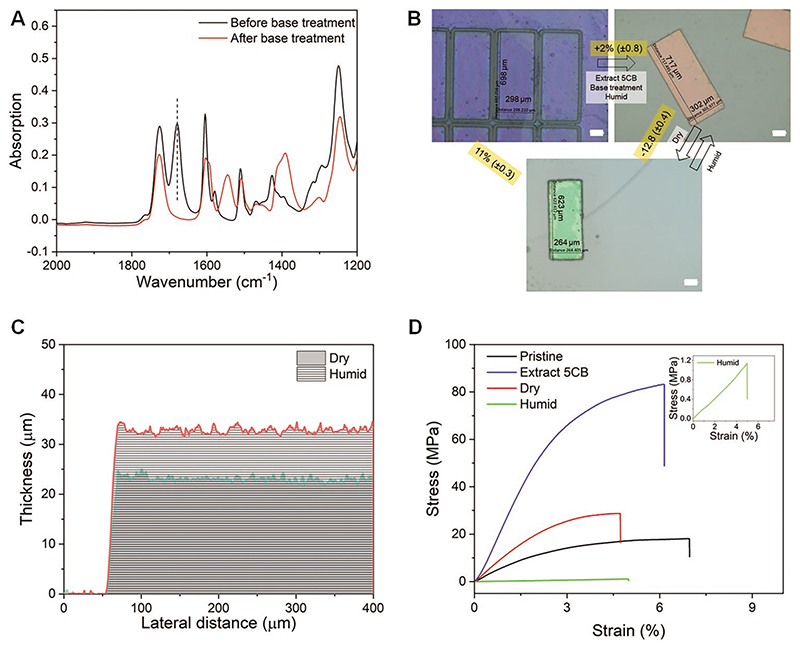
Characterization of the humidity-responsive CLCN. A) FTIR spectra of CLCNs before and after base treatment. The disappearance of the peak at 1680 cm^−1^ confirmed the breakdown of hydrogen bonding between carboxylic acid groups due to the deprotonation. B) Lateral size and color change of the CLCN. Scale bars: 100 μm. C) Thickness change of the CLCN film. D) Mechanical properties of the CLCN films in different states.

Next, we quantitatively study geometrical deformation and mechanical property change of the CLCN in response to humidity. After base treatment, the carboxylic acid groups were deprotonated, which was confirmed by the disappearance of the peak at 1670 cm^−1^ in FTIR spectra (Figure 2A). Geometrically, pristine CLCN micro flakes expanded slightly in the lateral direction by 2% (Figure 2B) with the color turning from blue to red in the humid state. Its thickness also changed from 24.9 μm (dry) to 34.6 μm (wet), as shown in Figure 2C. After drying, the CLCN shrank by 12.8% in the dry state compared to the humid state. Accompanied by geometrical change, the stiffness of LCN also shifted. Young's modulus increased from 670 to 1170 MPa after extraction of non-reactive 5CB since 5CB made the LCN a gel-like structure (Figure 2D). After base treatment and breaking hydrogen bonding between carboxylic acid groups, Young's modulus decreased to 320 MPa in a dry state; while Young's modulus shifted to 20 MPa upon absorption of water in humid conditions (experimental details in Experimental Section).

### 2.2 Colorful Actuator with Synergistic Shape Morphing and Color Change

**Figure 3:**
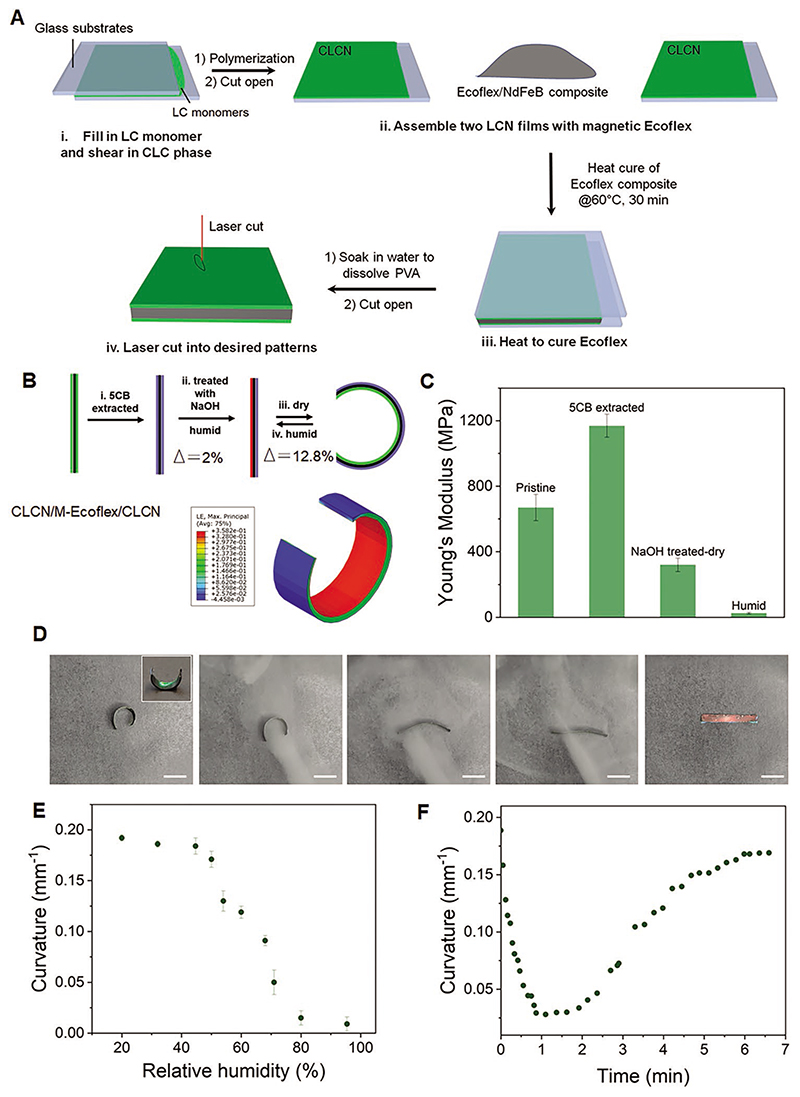
Synergistic shape morphing and color change of humidity-responsive colorful actuators. A) Fabrication of colorful CLCN/M-Ecoflex/CLCN actuator. B) Principle illustration of shape morphing of the colorful actuator. Humidity-induced swelling/shrinkage of asymmetric colorful actuators induced shape morphing. C) Young's modulus of the CLCN at different states. D) Humidity-induced shape and color change of colorful actuator stripe. Scale bar: 5 mm. E) Curvature as a function of the relative humidity. F) Deformation kinetics of the humidity-responsive actuator.

Here we employ cholesterics to break the coupling between actuation modes and cutting direction as the LC molecular long axis rotates along the helical axis in the thickness direction. The introduction of out-of-plane 3D actuation into structural colorful actuators provides opportunities for developing photonic devices with more degree-of-freedom. In detail, two CLCN films and magnetic Ecoflex (Ecoflex 00–30, Smooth-on Inc.) with NdFeB microparticles (MQP-15-7 with an average diameter of 5 μm, Magnequench) were processed to form a triple-layer structure with the magnetic Ecoflex layer in-between (Figure 3A). Firm interface bonding between LCN film and Ecoflex was ensured by a silane coupling agent (vinyltrimeth-oxysilane).^[45]^ Patterned film was harvested from the composite via laser cutting (LPKF ProtoLaser U3 Cutter).

Magnetic Ecoflex not only acts as a magnetically responsive layer for the driving force of magnetic actuation but also as a black contrast layer to enhance the color contrast by absorbing the light not subject to the Bragg reflection. This made the color of our actuators vivid without the need for additional black background as in previous reports. This strategy was inspired by and also observed in natural counterparts, e.g., the peacock spider *Maratus robinsoni*.^[10]^ To empower humidity-responsive actuation, asymmetry was introduced into the actuator via base treatment of only one side CLCN of the CLCN/Ecoflex/CLCN composite. During actuation, the hygroscopic LCN layer expanded when exposed to moisture and resulted in shape change. Experimentally, the colorful stripe (dimension of 2 mm × 20 mm) curled in response to moisture and the color also synergistically changed; after stopping the moisture flow, the strip recovered its initial curling state. Simulation results also match well with the experimental phenomena (Figure 3B–D). The film was more sensitive above the relative humidity threshold of 40%, and the response finished within a few minutes (Figure 3E,F).

**Figure 4:**
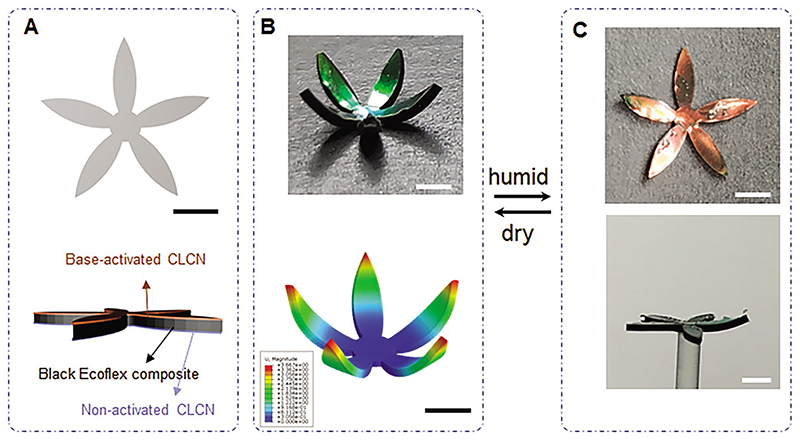
Humidity-induced synergistic shape morphing and color change of an artificial flower. A) Geometry and material design of the artificial flower. B) Green colorful flower in the dry condition and simulation result showing the curl shape of the base-treated flower. The color represents the displacement magnitude. C) In humid conditions, the artificial flower bloomed while its color changed from green to red. All scale bars: 3 mm.

The relatively low response rate was caused by water droplets condensed on the actuator surface in the actuation process of blowing moisture. The sensitivity and response rate could be improved in future work by using the electrospinning method to prepare microfibers with a larger area-to-volume value.^[46]^ Out-of-plane actuation of colorful actuators and the break of coupling between deformation mode and shape/pattern are shown with a five-petal artificial flower, which was base-treated on one side (Figure 4A). In the dry state after base treatment, the artificial flower was in a curling shape due to asymmetric base processing and shrinkage in the base treatment (Figures 3B and 4B), which was consistent with the simulation results. Upon exposure to moisture, the activated hygroscopic LCN layer swelled and expanded, causing the curly flower to “bloom” and turn flat with its color changing from green to red synergistically (Figure 4C; Movie S1, Supporting Information).

**Figure 5:**
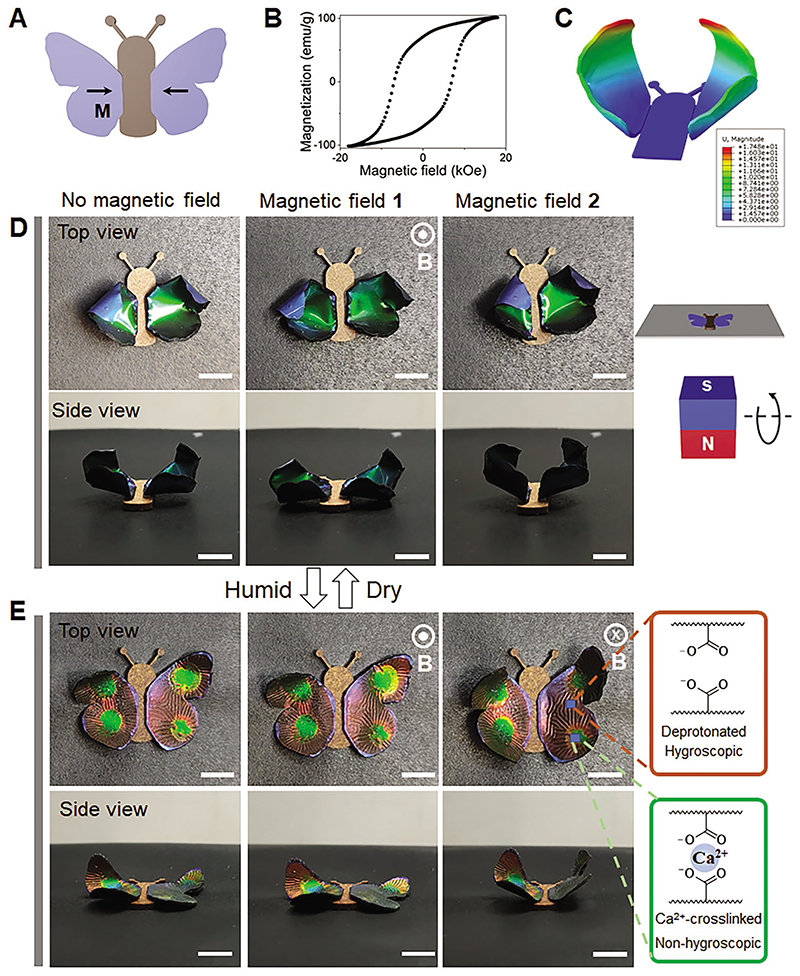
Control of the artificial colorful butterfly using humidity and magnetic field. A) Magnetization profile of the butterfly wings. B) Magnetic hysteresis loop of the magnetic actuator composite. C) Simulation showing the deformation of butterfly wings in dry conditions. The color represents displacement magnitude. D) Artificial butterfly in the dry condition and different magnetic fields were applied. Butterfly wings flapped in response to the magnetic field. E) Humidity-induced shape morphing and color change of butterfly wings in addition to wing flapping using the magnetic field. Hidden patterns were encrypted on butterfly wings by changing the hygroscopicity using calcium ions and the patterns were revealed upon humidity exposure. Scale bars in D and E: 5 mm.

Except for the passive synergistic color and shape change by the humidity, we also designed actuation using humidity and magnetic field. We developed an artificial butterfly that could flatter its wings in response to the magnetic field. The artificial butterfly's wings were attached to a non-responsive body and magnetized in opposite lateral directions (Figure 5A). The magnetic hysteresis curve is shown in Figure 5B. After base treatment, the treated side of the wings was hygroscopic. In dry conditions, the wings were of curling shapes in consistence with simulation results (Figure 5C). A permanent magnet (20 mm cubic N45 Neodymium magnet, MagnetMax) was used to actuate the butterfly wings. In an upward magnetic field perpendicular to the body plane, the wings oriented up; while the wings would be flattened when the magnetic field direction was set downward (Figure 5D).

In addition to magnetic actuation, the artificial butterfly was also responsive to humidity and could reveal encrypted patterns upon moisture stimulation. In the humid state, hygroscopic wings were hydrated, and the initially curled wings turned flat with color changing to red (Figure 5E; Movie S2, Supporting Information). The encrypted parts were patterned by treating desired parts with CaCl_2_, cross-linking carboxylate groups and making the CLCN less hygroscopic and relatively humidityinert. Moreover, the initially uniformly green butterfly wing surface becomes wrinkled due to different expansion coefficients of Ecoflex and CLCN.^[47]^ The artificial butterfly could be actuated by the magnetic field to change the orientation of butterfly wings (Figure 5E). The encrypted patterns on artificial butterfly wings would be useful for developing artificial insects with the functionality of deceiving predators, mimicking eyespot patterns of Peacock Pansy *Junonia almana*.

### 2.3 Motile Humidity Sensor

Monitoring humidity and providing parameters for humidity maintenance is of vital importance in many scenarios, e.g., egg hatching, since hatching productivity is highly related to relative humidity as well as temperature.^[48,49]^ However, for areas that locally generate moisture, humidity may be spatially non-uniform. Motile humidity sensors will be efficient for the flexible monitoring of humidity. Here we develop locomotive humidity sensors manipulated through the magnetic field and monitor the environmental humidity. We first demonstrate a motile sensor that could be used in non-confined space. The motile humidity sensor robot was made of three pairs of magnetic legs for locomotion. Legs of the same pair were magnetized in opposite directions and the neighboring legs were magnetized in opposite directions.

**Figure 6:**
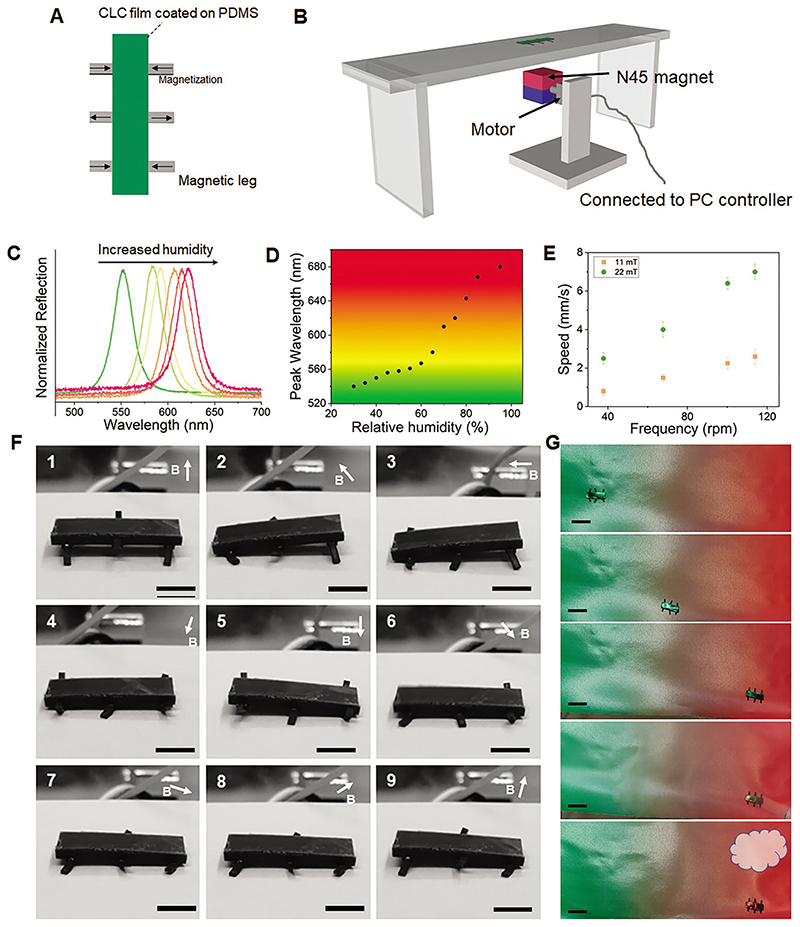
Walking robotic humidity sensor. A) Structure of the motile walking humidity sensor. A humidity-sensitive CLCN film was attached on the PDMS body with three pairs of magnetic legs. The arrows indicate the magnetization directions of legs. B) Schematic illustration of locomotion setup for the magnetic walking robot. The magnet rotation is controlled via an Arduino-programmed servo. C) Red-shift of CLCN reflection spectrum in response to increased humidity. D) The peak wavelength of the reflection spectra in different relative humidity. E) Robotic locomotion speed under different strengths and rotating speeds of the magnetic field. F) Snapshots of the locomotion process. Scale bar: 5 mm. G) Locomotion and humidity sensing of the motile humidity sensor. The initially green robot locomoted to another place under the actuation of magnetic field and changes color to red in response to varied humidity. Scale bar: 15 mm.

Structural colorful CLCN film was attached on the non-magnetic PDMS body for humidity sensing (Figure 6A; geometrical design in Figure S3, Supporting Information). A programmable rotating magnet (20 mm cubic Neodym N45 magnet) loaded on an Arduino-controlled servo (Parallax Feedback 360° servo) was used to provide a time-varying rotating magnetic field to steer the magnetic motile sensor (17 mm long) (Figure 6B, code in Supporting Information). The structural color was correlated with relative humidity (Figure 6C,D). The reflection band red-shifted with increasing relative humidity, making it possible to sense humidity via color recognition. Meanwhile, it was temperature-independent (Figure S4, Supporting Information), excluding the influence of temperature. Due to the comparable sizes between the magnet and artificial sensor, a non-negligible traveling wave of magnetic torque was exerted on different pairs of legs, resulting in the locomotion of the motile sensor (Movie S3, Supporting Information).

The locomotion speed was correlated with the strength of the magnetic field. By changing the distance of the permanent magnet from the walking robot, we tested the locomotion speed of the walking robot under different magnetic fields. The walking robot locomoted faster in a stronger magnetic field (Figure 6E) because of larger lifting and translational steps subjected to the magnetic field. The magnetic field strength would also vary while the magnet rotated and here, we used the strongest value as the reference. We also investigated the locomotion process of the robot (Figure 6F). When the magnetic field direction was upward, the front and rear magnetic legs lifted the robot; while when the magnetic field direction was downward, the middle pair of legs lifted the body. When the magnetic field was horizontal, magnetic legs oriented forward/backward sequentially. With the present new magnetization profile, the robot can be steerable with comparable speed compared with previous reports.^[38,50]^ When the robot locomoted to different places, it simultaneously was sensing the humidity by changing the body color (Figure 6G; Movie S3, Supporting Information). Due to the intrinsic angle-dependent property of the flat CLCN coating, the color needs to be read in normal incidence to obtain accurate results.

**Figure 7:**
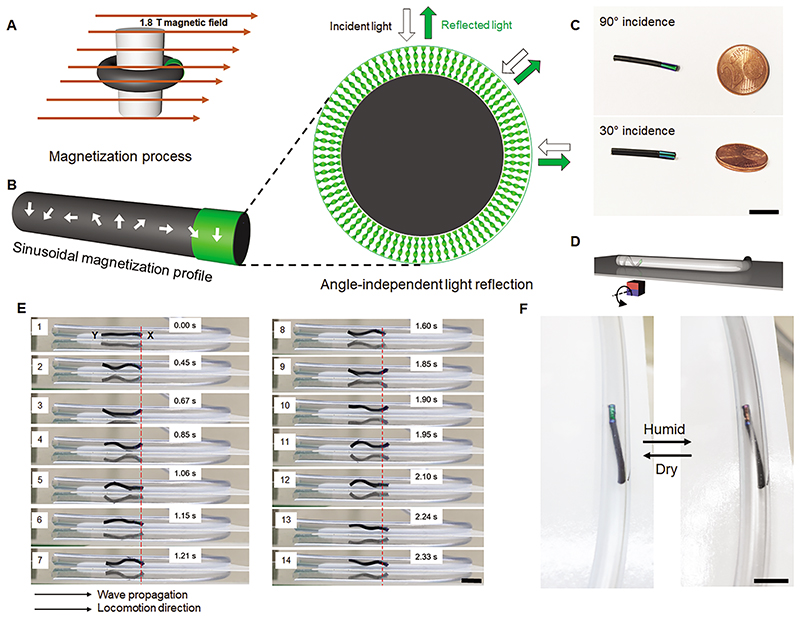
Cylindrical crawling humidity sensor for the confined tubular environment. A) Magnetization of the cylindrical colorful robot. B) Sinusoidal magnetization profile of the cylindrical robot and illustration of the viewing angle-independent reflection from the CLCN wrapped on the cylindrical robot. C) Images taken from different viewpoints show the viewing angle-independent reflection. D) Schematic illustration of the actuation setup for locomotion of cylindrical robot in a tube. E) Snapshots of crawling in a tube of the cylindrical robot. The undulation of the robot in the rotating magnetic field with the aid of friction contributes to the forward translational motion in the tube. F) Humidity-induced color change of the robot for humidity sensing in the confined tube. All scale bars: 10 mm.

We also developed a cylindrical motile humidity sensor for confined tubular spaces. Humidity is a key factor for some biological applications. For instance, control of humidity in small-volume bioreactors is of critical importance to prevent liquid evaporation, since liquid evaporation would cause relatively large volume change and result in an adverse impact on bioreactor operation.^[51]^ Here we integrated a wirelessly controlled magnetic robot with a humidity CLCN sensor to develop a motile sensor that could be used in confined spaces where commercial humidity sensors are difficult to implement. In detail, a cylindrical magnetic robot made of magnetic Ecoflex was inscribed with a sinusoidal magnetization profile by wrapping the robot (diameter 2 mm, length 25 mm) into a circular shape with the aid of a fixture (Figure S5, Supporting Information) when being magnetified in a strong magnetic field (magnetic field strength: 1.8 T, Figure 7A,B). The CLCN was wrapped at one end of the cylindrical robot as a humidity sensor, while the motile cylindrical robot enabled the locomotion of the integrated system. As the CLCN film followed the circular contour, the helical axis of cholesterics was constantly in the radial direction. The reflection was thus viewing angleindependent (Figure 7B). The sensor displayed the same color when viewed at the incidence angle of 90° and 30° (Figure 7C). We then tested the locomotion capability of the cylindrical robot in a confined tube (inner diameter 5 mm). The system's locomotion was achieved with a rotating magnetic field provided by the rotating permanent magnet underneath the tube (Figure 7D).

The cylindrical robot was able to crawl in the tube due to friction between the robot body and the tube wall. In detail, the crawling was achieved via sequential contracting and stretching of the body project length in the rotating magnetic field.^[52]^ Here the cylindrical robot needed not to form a helical shape, different from the sheet robot in our previous report.^[52]^ The robot first formed a wave shape in response to the magnetic field (Figure 7E2), with the Y end contacting and anchoring to the upper wall. As the magnetic field rotated, the robot stretched its project length by keeping the Y end anchoring to the upper wall while moving the X end from the lower wall to the upper wall (Figure 7E3), causing a forward translation of the X end. During this process, trough of the body wave was transmitted toward X end.

As the magnetic field further rotated, Y end detached from the upper wall, and the robot contracted the body by forming a wave shape, reducing the projection length and causing a forward translation of the Y end (Figure 7E4). After Y end anchored to the lower wall (Figure 7E5), the robot formed a wave shape that horizontally mirrored the wave shown in Figure 7E2, which initiated contracting/stretching of the body and the robot forward translation in the following phases (Figure 7E5,E6). With such concertina-like crawling locomotion, the robot moved toward the X end (Figure 7E7–E14). In this process, the wave propagation direction was consistent with the locomotion direction. With this motile robot, the humidity sensor was able to be deployed deep into the tube. It could also pass through a tube with a turning angle of up to 90° by changing the magnetic orientation (Movies S4 and S5, Supporting Information). This is an advantage over ordinary commercial humidity sensors that are bulky and tethered. The humidity change in the tube can be reflected by the color change of the sensor (Figure 7F).

## 3 Conclusion

We have developed magnetic colorful actuators and motile sensors. The magnetic colorful actuators could be controlled by humidity and magnetic field, and exhibited synergistic shape morphing and color change. In addition, the humidity-sensitive CLCN was integrated with magnetically powered locomotion to develop motile humidity sensors, which could be used in monitoring humidity variation in different places even in confined tubular scenarios. In future work, the sensitivity of actuators and sensors will be improved to develop colorful actuators and sensors with a quicker and more sensitive response. Our research would be valuable for developing wirelessly controlled actuators and sensors with vision-based read-out results.

## 4 Experimental Section

### Materials

LC monomers 1 (4-(3-(acryloyloxy)propoxy)benzoic acid), 2 (4-((6-(acryloyloxy)hexyl)oxy)benzoic acid), 3 (4-methoxyphenyl 4-((6-(acryloyloxy)hexyl)oxy)benzoate, synonym RM105) and 5 ((3R,3aS,6S,6aS)-hexahydrofuro[3,2-b]furan-3,6-diyl bis(4-((4-(((4-(acryloyloxy)butoxy)carbonyl)oxy)benzoyl)oxy)benzoate), synonym LC756) were obtained from Synthon Chemicals. LC monomer 4 (2-methyl-1,4-phenylene bis(4-(3-(acryloyloxy)propoxy)benzoate), synonym RM257) was purchased from Wilshire Technologies company. Chemical 6 (4-Cyano-4'-pentylbiphenyl, synonym 5CB), photoinitiator 7 (IRG369), inhibitor 8 (butylated hydroxytoluene), and poly(vinyl alcohol) (Mw 85–124 kDa) were purchased from Sigma-Aldrich. NdFeB microparticles (MQP-15-7, with an average diameter of 5 μm) were purchased from Magnequench.

### CLCN Preparation

Glass substrates were cleaned in acetone and n-isopropanol with ultrasonication for 10 min, and then treated with UV ozone for 20 min. PVA solution (3% PVA in water) was spun coated onto substrates at a speed of 1500 rpm for 45 s and then dried on a 120 °C hotplate for 30 min. The PVA-coated glass substrates were then rubbed with a cloth and assembled into liquid crystal cells with 20 μm spacers to determine the cell gap. The LC mixture consists of compounds 1–8 with the mass ratio of compound 1 : 2 : 3 : 4 :5 : 6 : 7 : 8 = 25 : 25 : 15 : 12 : 7 :15 : 1 : 0.5. The ratio of monomers was adjusted carefully to have a proper temperature range of cholesteric phase from 22 to 71 °C (DSC curve in Figure S1, Supporting Information) for experimental processing while maintaining the mechanical strength of the film and sensitivity to humidity. The ratio of monomers could be adjusted according to needs, for instance, the ratio of monomers 1 and 2 could be increased to have more humidity sensitivity while sacrificing some extent of the temperature processing window of the monomer mixture; the concentration of chiral dopant 5 could be adjusted to afford different structural color in the initial state. The LC monomer mixture in the isotropic phase at 80 °C was filled into the LC cell by capillary force, and then slowly cooled down to 37 °C. After gentle mechanical shear, the LC cell exhibited vivid reflection color and then photopolymerization under UV light for 20 min with a following post-cure at 120 °C for 10 min.

### Preparation of CLCN/Magnetic Ecoflex/CLCN Composite

After polymerization, the LC cell was carefully cut open using a razor blade with the LCN film sticking on one glass substrate. The LCN was first processed with air plasma (75 W, 2 min) and then treated with vinyltrimethoxysilane (2% in methanol) via spin-coating at a speed of 1500 rpm for 45 s. Then magnetic Ecoflex0030 composite (mass ratio of Ecoflex part A : part B : NdFeB microparticles : vinyltrimethoxysilane = 100 : 100 : 200 : 2) was sandwiched between two LCN films. The gap (thickness of magnetic Ecoflex) between LCN films was 300 μm determined using suitable spacers. The magnetic Ecoflex was cured at 95°C for 30 min. The triple-layered composite was carefully harvested from the glass substrate after soaking in water to dissolve the sacrificial PVA layer. LCN/Ecoflex/LCN sheets with predesigned shapes were fabricated with a laser cutter (LPKF ProtoLaser U3 Cutter).

### Fabrication of Locomotive Humidity Sensor: Fabrication of Walking Robot

The walking robot robotic sensor was composed of three pairs of magnetic Ecoflex legs with a CLCN-coated PDMS body. Ecoflex (Part A + Part B) was thoroughly mixed with magnetic NdFeB microparticles, degassed in a vacuum chamber for 10 min, poured onto a plastic substrate and blade-coated into a film with a thickness of ≈300 μm, which was cured at 45 °C for 4 h. Magnetic legs were laser cut from the magnetic Ecoflex film and magnetized in the designed direction using MicroSense EZ7 vibrating sample magnetometer. To prepare the PDMS body, CLCN was plasma treated and coated with silane to form firm bonding with PDMS. A proper amount of PDMS (mass ratio of base: curing agent = 10:1) mixed with black dye (Silc Pig™, Smooth-on Inc., 1.8 wt.% of PDMS) was poured onto the CLCN film sticking on a glass substrate. After curing in a 90 °C oven for 3 h, the composite was immersed in water to release the composite from the glass substrate by dissolving the PVA layer from the CLCN preparation step. To assemble the magnetic robot, the magnetic legs were glued onto the PDMS body with a small amount of Ecoflex as the glue.

### Fabrication of Cylindrical Robot

To fabricate the cylindrical magnetic robotic humidity sensor, uncured magnetic Ecoflex (Ecoflex: magnetic microparticles = 1:1 in mass) was injected into a plastic tube (inner diameter: 2 mm) and cured in a 60 °C oven for 4 h. After curing, the cylindrical magnetic Ecoflex was carefully pulled out from the plastic tubular mold. The cylindrical magnetic Ecoflex composite was cut into 3.5 mm long pieces and magnetized with a sinusoidal magnetization profile by confining the cylindrical Ecoflex rod in a circular fixture (Figure S5, Supporting Information) when magnetized with MicroSense EZ7 vibrating sample magnetometer (VSM).

### Characterization of Liquid Crystal Monomers and Polymers

Liquid crystalline phase transition temperature was measured with DSC curves using a differential scanning calorimeter (DSC 2500, TA Instruments) with a temperature ramp rate of 10 °C min^−1^. Cholesteric textures of LC samples were characterized with Zeiss AXIO polarized optical microscope. Reflection spectra were measured with PerkinElmer Lambda 1050+ photospectrometer and Thorlabs CCS200/M Compact Spectrometer. Keyence laser scanning microscope VK-X250 was used to measure the sample thickness in dry/humid states. The mechanical properties of samples were measured with a tensile machine Instron 5942 using a 10 N load cell with a displacement rate of 0.05 mm s^−1^. In the tensile test, CLCN films in the size of 10 mm × 20 mm were used. The thickness of films at different states was measured for the calculation of corresponding Young's moduli.

### Simulation

Finite element analysis (FEA) was performed using commercial software (ABAQUS/Standard, version 2020, Dassault Systèmes®) to verify the deformation of the LCN/magnetic Ecoflex/LCN sandwich structure. Eight node hexahedral elements with reduced integration (C3D8R) were used to model the structures and included at least 8 elements along the width to accurately capture the bending deformation. The swelling of the LCN was predicted by using the analogy to thermal expansion. Orthotropic thermal expansion properties were defined for the LCN material and a temperature differential was applied to induce the swelling. The thermal expansion constants were found by experimentally measuring the shape change of LCN samples. Both LCN and magnetic Ecoflex were modeled as linear elastic materials and the elastic constants were found experimentally. The change in elastic modulus of LCN material due to NaOH treatment was incorporated by defining a temperature-dependent Young's modulus.

### Characterization of Humidity-Response

A commercial humidifier was used to blow moisture to the humidity-responsive actuator. To investigate the responsive rate, moisture was applied to the colorful stripe in Figure 3 as the moistening process, then the colorful stripe was left in the open air to dry to observe the recovery process (Figure 3F). To characterize the curvature in response to different relative humidity, the colorful strip and a hygrometer were placed inside a plastic transparent box with an opening. Moisture was blown into the box until the hygrometer number reached 98%. The curvature of the colorful stripe was recorded. Then the opening of the box was opened and the relative humidity of the box decreased gradually. The decrease rate of the relative humidity was ≈1% per minute until reaching equilibrium with the indoor relative humidity at ≈25%. During this process, curvatures of the colorful stripe were recorded at different relative humidity.

## Supplementary Material

Supplemental Materials

Supplemental Movie 1

Supplemental Movie 2

Supplemental Movie 3

Supplemental Movie 4

Supplemental Movie 5

## Data Availability

The data that support the findings of this study are available from the corresponding author upon reasonable request.
