# Supplementary material for: Cholesteric Liquid Crystal Polymeric Coatings for Colorful Artificial Muscles and Motile Humidity Sensor Skin Integrated with Magnetic Composites
Source: Adv Funct Mater. Author manuscript; Available in PMC 2023 Jun 8. (PMC7614630; doi:10.1002/adfm.202300731)
Supplement: Supplemental Materials [file EMS176199-supplement-Supplemental_Materials.pdf]

ADVANCED FUNCTIONAL MATERIALS

Supporting Information

for *Adv. Funct. Mater.,* DOI: 10.1002/adfm.202300731

Cholesteric Liquid Crystal Polymeric Coatings for Colorful Artificial Muscles and Motile Humidity Sensor Skin Integrated with Magnetic Composites

*Wei Feng, Aniket Pal, Tianlu Wang, Ziyu Ren, Yingbo Yan, Yanqing Lu, Huai Yang, and Metin Sitti**

Supporting Information

Cholesteric liquid crystal polymeric coatings for colorful artificial muscles and motile humidity sensor skin integrated with magnetic composites

*Wei Feng, Aniket Pal, Tianlu Wang, Ziyu Ren, Yingbo Yan, Yanqing Lu, Huai Yang, Metin Sitti**

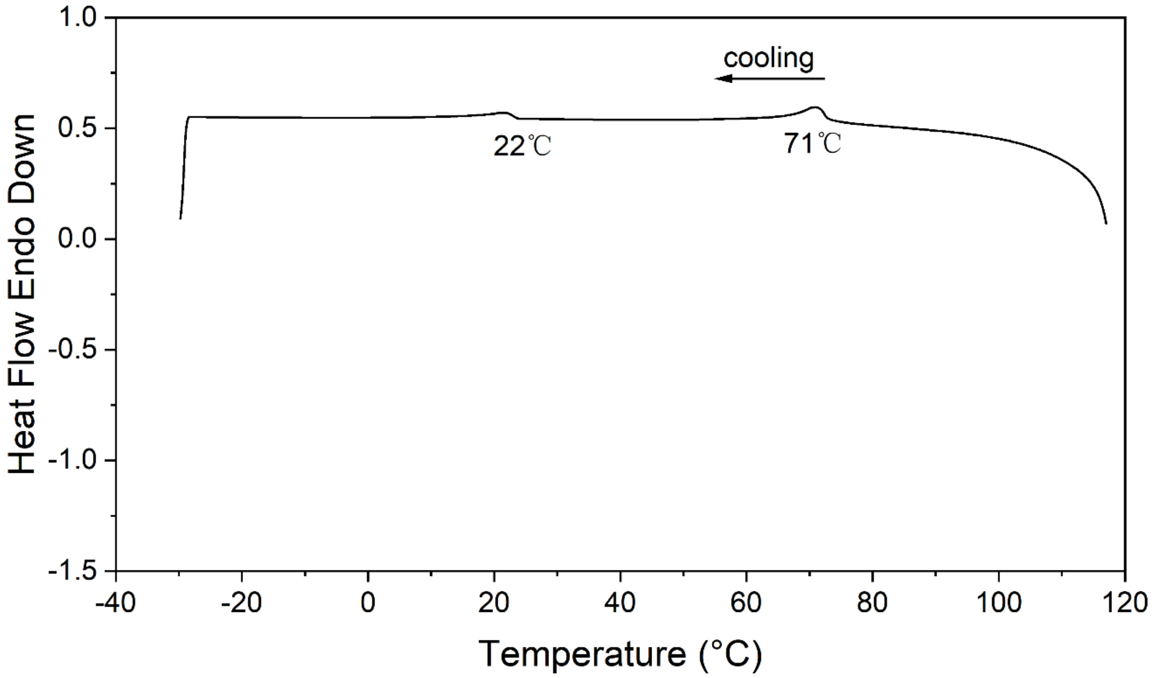

Figure S1. Cooling differential scanning calorimetry (DSC) thermogram of the LC monomer mixture. The isotropic-to-nematic transition temperature $T_{I\text{-}N}$ and nematic-to-crystal transition temperature $T_{N\text{-}Cry}$ were 71°C and 22°C, respectively.

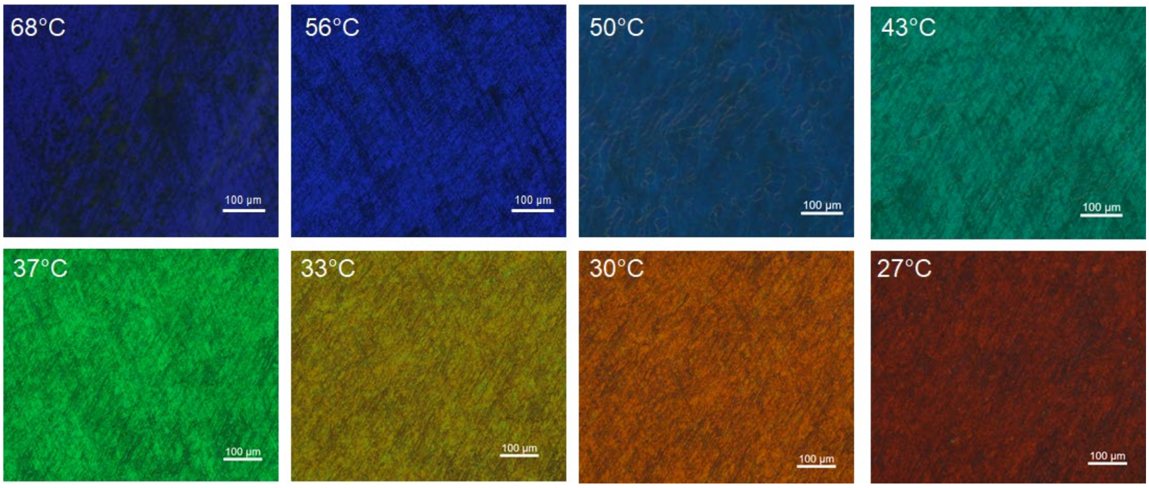

Figure S2. Reflectance of cholesteric monomer mixture at different temperatures. The reflection color red-shifted upon cooling. The initial color of polymerized CLCN could be controlled by choosing a suitable polymerization temperature.

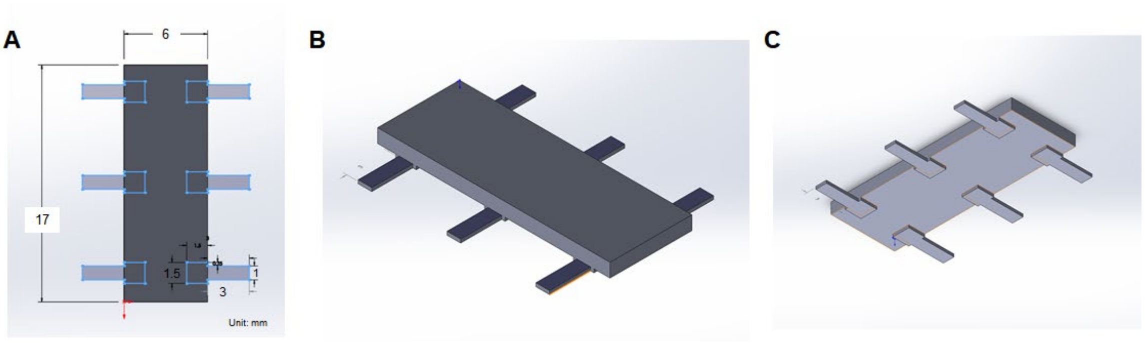

Figure S3. Geometric parameters of the walking robot.

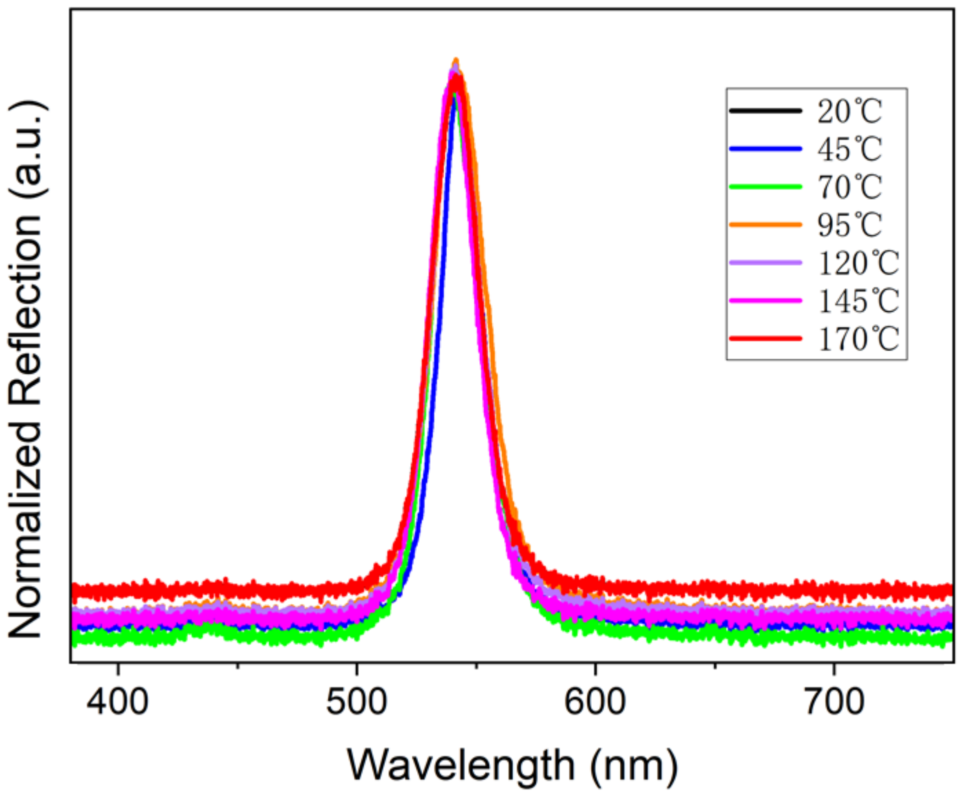

Figure S4. Reflection of the present CLCN was stable at different temperatures. The chromic independence of temperature is due to the dense crosslinking of the polymeric network.

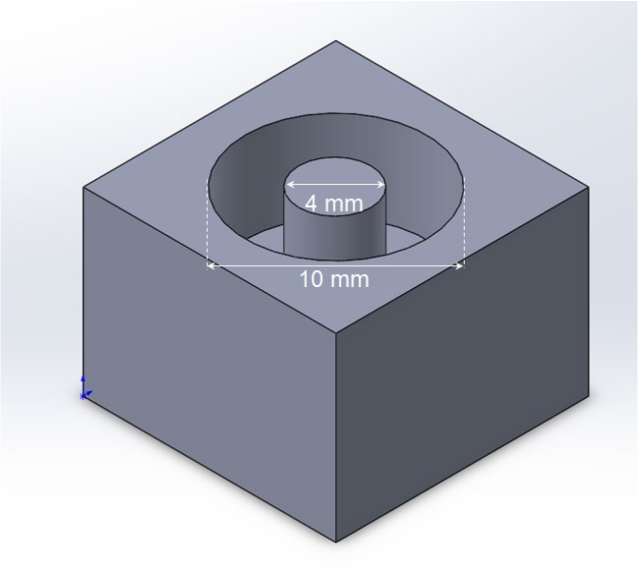

Figure S5. Geometric design of the fixture for magnetization of the cylindrical robot in Figure 7 in the main text.

Supplementary code for controlling the servo using Arduino

Arduino UNO microcontroller board was combined with a Parallax Feedback 360° High Speed Servo to rotate the magnet.
The following code was used to control the servo:

```
#include <Servo.h>    // Use Servo library, included with IDE
Servo myServo;        // Create Servo object to control the servo
// when the value is smaller than 90 -- clockwise rotation
// when the value is larger than 90 -- counter-clockwise rotation
double speed_write =120;
void setup() {
  myServo.attach(9);  // Servo is connected to digital pin 9
}
void loop() {
  myServo.write(speed_write);
}
```

Supporting Movies

Movie S1. Synergistic shape and color change of the colorful artificial flower. The five-pedal shape flower with a sandwich structure hygroscopic CLCN/Ecoflex/unactivated CLCN showed humidity-responsive flower blooming, and its color synergistically turned from green to red.

Movie S2. Humidity-responsive synergistic color and shape morphing of the artificial butterfly. Upon exposure to moisture, the base-treated CLCN layer of the artificial butterfly wing expanded, turning the initially curling shape to flat, and the cholesteric pitch simultaneously expands with its reflection band turning red. The encrypted patterned areas remained intact and green, revealing the encrypted patterns.

Movie S3. Motile walking robot for humidity sensing. The locomotion of the walking robot was actuated by a rotating magnetic field. The motile robot could locomote to different places for humidity sensing.

Movie S4. Locomotion of motile cylindrical humidity sensor (side view). The cylindrical robot with the humidity sensor could locomote in a confined tubular environment for humidity sensing. The locomotion was powered by a rotating magnetic field.

Movie S5. Locomotion of motile cylindrical humidity sensor (top view). The robot can locomote in a curved tube by changing the orientation of the rotating permanent magnet.
